# Optimization of the Cytotoxic Activity of Three* Streptomyces* Strains Isolated from Guaviare River Sediments (Colombia, South America)

**DOI:** 10.1155/2018/2839356

**Published:** 2018-07-19

**Authors:** Laura Ramirez-Rodriguez, Boghos Stepanian-Martinez, Maria Morales-Gonzalez, Luis Diaz

**Affiliations:** Facultad de Ingenieria, Universidad de La Sabana, Campus del Puente del Comun, Km 7 Autopista Norte de Bogotá, Chia, Colombia

## Abstract

Three* Streptomyces *strains isolated from Guaviare sediments (Colombia, South America) with cytotoxic activity against prostate cancer (PC3), breast cancer (MDA-MB-231), and lung cancer (A549) line cells were studied. The present investigation reveals the enhancement of the cytotoxic activity evaluating different values of pH, carbon sources (sucrose, glucose, xylose, maltose, and starch), and nitrogen sources (malt extract, yeast extract, meat extract, peptone, and potassium nitrate). Based on the response surface methodology, the isolates* Streptomyces aburaviensis* (73) had the maximum activity for lung cancer (IC_50_= 25.00 ± 1.86 ppm) with 4% of yeast extract, 3% of starch, and a pH value of 7.* Streptomyces gramineus* (386) had the maximum activity against prostate cancer (IC_50_= 6.14 ± 2.07 ppm) with 5% of malt extract, 3% of glucose, and a pH value of 6. Finally,* Streptomyces psammoticus* (519) had the maximum activity against breast cancer (IC_50_= 35.53 ± 2.71 ppm) with 1% of yeast extract, 4% of starch, and a pH 8. The results suggest that the ethyl acetate extracts from isolates* Streptomyces aburaviensis* (73),* Streptomyces gramineus* (386), and* Streptomyces psammoticus* (519) have a potential for use in pharmaceuticals as cytotoxic agents.

## 1. Introduction

Cancer is a disease that has great relevance today because it is one of the most serious human health problems in the world [1]. In 2018, 1,735,350 new cancer cases and 609,640 cancer deaths are projected to occur in the United States [2]. The most common types of cancers in 2017 were prostate with 19% and lung and bronchus cancer with 14% for men [3]. Siegel et al. [2] state that, for women, the most commonly diagnosed cancers are breast, lung and bronchus, and colorectum; which collectively represent one-half of all cases. The lifetime probability of being diagnosed with invasive cancer is higher for men (39.7%) than for women (37.6). The American Cancer Society states that cancer occurs when there is uncontrolled cell growth and spread of abnormal cells caused by external factors, such as inherited genetic mutations, hormones, and immune conditions [3]. Cancer is treated with surgery, radiation, chemotherapy, hormone therapy, immune therapy, and targeted therapy [4]. Chemotherapy is one of the most effective antitumor treatments, but this therapy needs a new active compound as the major sources of natural products with anticancer activity are microbes [5]. Maskey et al. indicate that* Actinobacteria* is famous as a great source of antitumor compounds, as cited in [6], mainly* Streptomyces* sp. There are several anticancer drugs derived from* Streptomyces* sp. like Proximicin A, B, and C, Daunomycin, Drimentine G, Indotertine B, Bleomycin, and Benzastatins that are already in clinical use [6].* Streptomyces* spp. are Gram positive filamentous, sporulating bacteria with DNA rich in G+C content [7]; they have been isolated in major part from soil and aquatic habitats like rivers which have been poorly explored as a source of* Streptomyces* spp. with biological activity (antibacterial, antifungal, antitumor, and antioxidant) [8]. These bacteria are the major source of bioactive compounds since they shelter different gene clusters, thus producing different compounds with diverse chemical backbones [9]. Most of these compounds are secreted in the broth and can be extracted with organic solvents [10]. The aim of this study is to investigate the cytotoxic activity (against PC3, MDA-MB-231, and A549 cancer cell lines) of extracts from* Streptomyces* broths isolated from a Colombian river, establishing the best conditions of carbon source, nitrogen source, and pH.

## 2. Materials and Methods

### 2.1. *Streptomyces* Strains and Their Maintenance

A total of forty Actinobacterial strains were selected based on their antimicrobial activity from the previously described collection of 275 isolates collected from two different locations in the Guaviare river, Colombia (2°34′51.6′′N 72°41′34.7′′W and 2°34′51.4′′N 72°39′53.6′′W) [8]. Morphological parameters such as aerial and substrate mycelium arrangements were carried out on ISP 3 medium as described in Bergey's Manual of Systematic Bacteriology [11] and by Shirling and Gottlieb [12]. The spore mass and mycelium of the pure isolate were stored at −80°C as glycerol suspension (40%, v/v) for further analysis.

### 2.2. Preparation of Active Organic Extracts

*Streptomyces* strains were inoculated in 50 mL Erlenmeyer flask containing 10 mL of ISP 2 (8.0 g of glucose, 10.0 g of malt extract, and 4.0 g of yeast extract, pH 7.2 ± 2°C) for seven days at 30°C under continues shaking (200 rpm) [13], and the medium was sterilized by autoclaving at 121°C for 15 min prior to experiment [14]. Further, equal volume (1:1) of ethyl acetate was added and mixed by vigorous shaking (200 rpm). The organic phase was collected, washed with three volumes of water, and evaporated to dryness. The extract was suspended in phosphate buffered saline (PBS) at a concentration of 1000 ppm [15, 16].

### 2.3. Cytotoxicity Assay

Human A549 lung adenocarcinoma cancer cell line (ATCC® CCL-185), PC3 adenocarcinoma prostate cancer cell line (ATCC CRL-7934), and L929 mouse fibroblasts (ATCC CRL-6364) were grown as monolayer culture in DMEM medium (10% (v/v) with fetal bovine serum, 1% (v/v) penicillin, and 1% (v/v) streptomycin). The breast cancer cell line MDA-MB-231 (ATCC HTB-26) was incubated in RPMI (10% (v/v) with fetal bovine serum, 1% (v/v) penicillin, and 1% (v/v) streptomycin). The cells were maintained in a humidified atmosphere with 5% CO_2_ at 37°C [17]. Briefly, 100 *μ*L of cell suspension was inoculated to 96-well plates with a density of 5 × 10^3^ cells/well and cultured for 24 h. After that, culture medium was replaced with 100 *μ*L serum-free medium containing various concentrations (10, 25, 50, 100, and 200 ppm) of* Streptomyces* broth extracts, each extract was tested in triplicate based on Ravikumar et al. [16]. Doxorubicin (1-25 ppm) was used as the cytotoxic positive control, medium supplemented with bovine serum albumin 1% (w/v) as negative control (100% survival), and free medium containing PBS as blank [18].

The cell viability was assessed by adding 10 *μ*l of MTT (5 mg/mL) per well, after 48 h of incubation, and the plates were incubated with 5% of CO_2_ at 37°C for 3 h. The formazan crystals formed in the cells were dissolved with 100 *μ*L of DMSO measured at 570 nm using a microplate reader (iMark^TM^ Microplate Reader) [19]. Cytotoxicity of each sample was expressed as an IC_50_ value. The IC_50_ value is the concentration of the test sample that causes 50% inhibition of cell growth averaged from three replicate experiments [15].

### 2.4. Effect of Different Carbon and Nitrogen Sources on Cytotoxic Activity

To improve the cytotoxic activity of tree* Streptomyces *strains with the highest cytotoxic activity (73, 386, and 519), preliminary experiments of carbon and nitrogen sources were carried out.* Streptomyces* strains were grown in 12 well microplates with 5 mL basal agar ((NH_4_)_2_SO_4_ 2.64 g/L, KH_2_PO_4_ 2.38 g/L, K_2_HPO_4_ 4.31 g/L, MgSO_4_ x 7H_2_O 1.0 g/L) and 1.0 ml/L trace element solution (CuSO_4_ x 5H_2_O 0.64 g/L, FeSO_4_ x 7H_2_O 0.11 g/L, ZnSO_4_ x 7H_2_O 0.15 g/L, MnCl_2_ x 4H_2_O), supplemented with 0.8% (w/v) of carbon source: lactose, galactose, ribose, xylose, maltose, starch, sucrose, fructose, and carboxymethylcellulose, with glucose as positive control and no carbon source as negative control [8]. Nitrogen sources were evaluated in the same way at the same conditions at 1.4% (w/v): L-asparagine, L-glutamine, proline, ammonium sulphate, meat extract, yeast extract, potassium nitrate, peptone, and malt extract [18]. The experiments were performed for 7 days, pH 7, 200 rpm, and 30°C; the results demonstrated that the five carbon sources that enhanced the cytotoxic activity of* Streptomyces* strains were glucose, xylose, maltose, starch, and sucrose. Likewise, the nitrogen sources selected were meat extract, yeast extract, potassium nitrate, peptone, and malt extract [20].

### 2.5. Media Optimization

The influence of environmental and nutritional conditions in growth culture, such as initial pH (6–8) and carbon and nitrogen sources concentrations (0,1-5% w/v) [21], on the cytotoxic activity was evaluated using a face central composite design (FCCD), evaluating 15 trials with five levels for each factor.

The experimental results of FCCD were statistically studied by analysis of variance (ANOVA). All the experiments were done in triplicate, and the average of the cytotoxic activity was obtained and taken as the dependent variable or response (*Y*). The results were fitted via the response surface regression procedure using the following second order polynomial equation [22]:

(1) $$\begin{array}{c} Y=\beta_{0}+\overset{n}{\underset{i=1}{\sum }}\beta_{i}x_{i}+\overset{n-1}{\underset{i=1}{\sum }}\overset{n}{\underset{j=i+1}{\sum }}\beta_{ij}x_{i}x_{j}+\overset{n}{\underset{i=1}{\sum }}\beta_{ii}{x_{i}}^{2} \end{array}$$

in which *Y* is the predicted response, *β*_0_ is the regression coefficients, *β*_*i*_ is the linear coefficient, *β*_*ii*_ is the quadratic coefficients, *β*_*ij*_ is the interaction coefficients, and *Xi* is the coded levels of independent variables [20].

### 2.6. Molecular Characterization

Total genomic DNA was isolated using the phenol chloroform method [23]. PCR amplification of 16S rRNA gene locus was conducted using primers 27F (5′-AGAGTTTGATCMTGGCTCAG-3′) and 1492R (5′- TACGGYTACCTTGTTACGACTT-3′). The PCR product was detected by agarose gel electrophoresis [8]. PCR thermal cycling is described elsewhere [24]. PCR products were sequenced by Macrogen (Korea). Sequences were assembled using the CLC Main Workbench 7.0 program and were aligned using SINA. The sequences were subjected to homology search using EzTaxon system (https://blast.ncbi.nlm.nih.gov/Blast.cgi) and the BLAST program of the National Centre for Biotechnology Information (NCBI) [18].

### 2.7. LC-MS Analysis

The dry extracts were dissolved in methanol and were subjected to LC-MS analysis on LCMS-2020 (Shimadzu Corp, Japan) system equipped with a single quadrupole analyser and an electrospray ion source (ESI). The analysis was done using a Synergi RP C_18_ column (Phenomenex) dimension 150 × 4.4 mm and film thickness of 4 *μ*m (0.7 mL/min flow rate; 30°C column oven temperature). 0.1% formic acid in acetonitrile and 0.1% formic acid in water were used as mobile phases A and B, respectively. The gradient elution was done as follows: 0 min, 10% A; 3 min, 10% A; 14 min, 40% A; 21 min, 70% A; 25 min, 100% A; 30 min, 10% A. ESI was simultaneously operated in positive and negative ion mode (scan 200–800 m/z; 250°C CDL temperature; 1.2 kV detector voltage; 1.3 L min−nebulizing gas flow rate; 8.0 L min−drying gas flow rate) [25].

### 2.8. Statistical Analysis

Design Experts® v7 software was used for the experimental designs and the models were statistically studied by analysis of variance (ANOVA) applied to the established regression, with a statistical significance (P<0.05). The surface plots were made with Statistica ® software.

## 3. Results

Forty Actinobacterial extracts at 100 ppm were tested in the cytotoxic assay against prostate (PC3), lung (A549), and breast (MDA-MB-231) cancer lines (Table 1).* Streptomyces* strains 73, 386, and 519 presented the highest cytotoxic percent so that values of IC_50_ (Table 2) were calculated to improve their cytotoxic activity.

**Figure 1:**
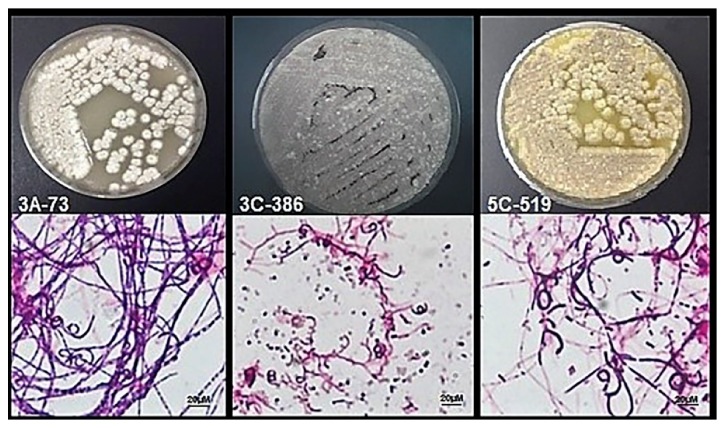
Morphologic features and Gram stain of* Streptomyces* strains with cytotoxic activity grown in ISP 3 agar medium.

The* Streptomyces* strains 73, 386, and 519 were characterized based on colony, morphology, mycelium coloration, and pigment diffusion. The light microscopy observation of isolates on ISP 3 media showed a straight chain section with no fragmentation of the aerial mycelium. The isolates are Gram positive strains and presented spores surfaced oval shaped with short compact spirals indicating that they belong to the genus* Streptomyces*. [26]. Consequently,* Streptomyces* 73 showed an aerial and substrate mycelium, white and grey, respectively.* Streptomyces* 386 and 519 showed a white aerial mycelium and brown substrate mycelium with a pigment production similar to melanin (Figure 1).

### 3.1. Taxonomical Identification

**Figure 2:**
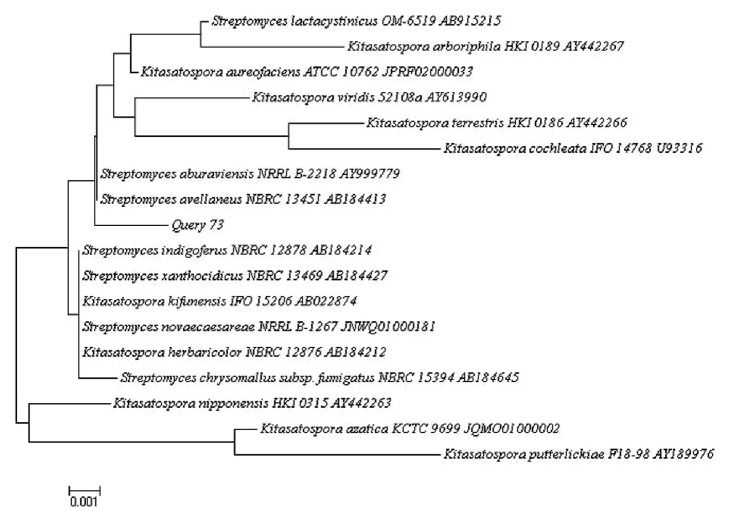
Neighbor-Joining phylogenetic tree based on 16S rRNA gene sequence showing the relationship between strain 73 and representatives of some other related taxa.

**Figure 3:**
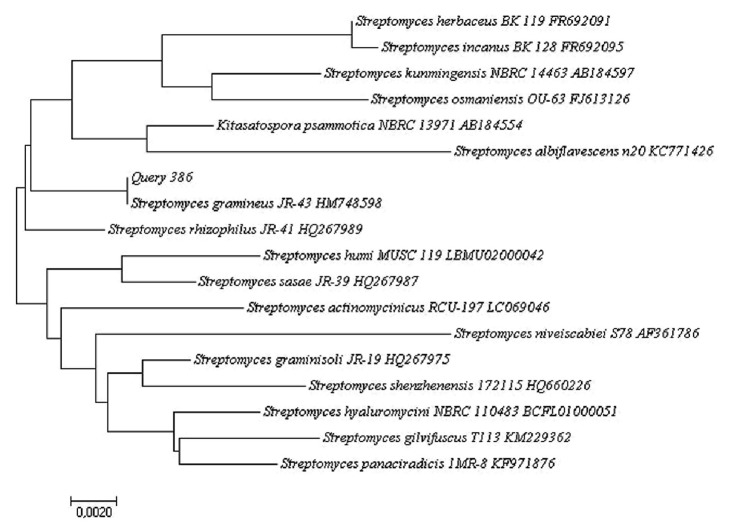
Neighbor-Joining phylogenetic tree based on 16S rRNA gene sequence showing the relationship between strain 386 and representatives of some other related taxa.

**Figure 4:**
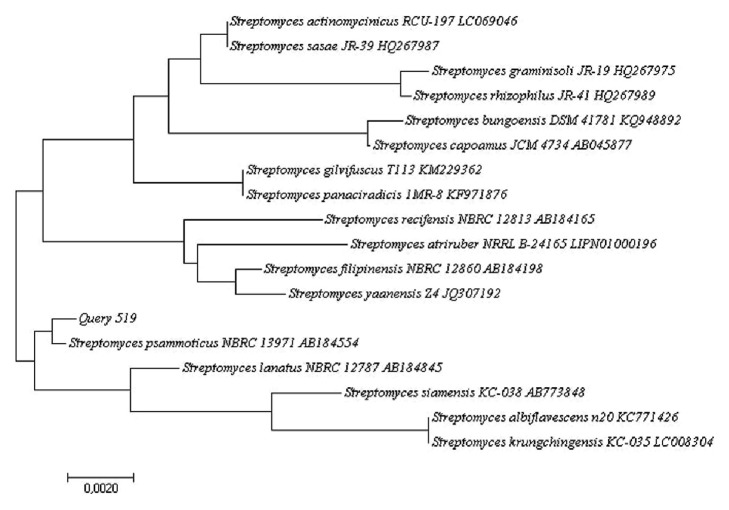
Neighbor-Joining phylogenetic tree based on 16S rRNA gene sequence showing the relationship between strain 519 and representatives of some other related taxa.

The phylogenetic data of the 16S rRNA gene sequences of three isolates were obtained aligning the nucleotides, and the representative sequences of related type strains of the genus* Streptomyces *were retrieved from the GenBank/EMBL/DDBJ databases using CLUSTAL-X software [27]. Phylogenetic trees were constructed with the Neighbor-Joining method [28] (Figures 2, 3, and 4) using MEGA version 6.0 [29]. The EzTaxon system (http://www.ezbiocloud.net/eztaxon) was used to calculate sequence similarities [30]. Considering that the most recent definition of a prokaryotic species based on 16S rRNA gene sequence similarity is determined by a minimum similarity of 98.7% [31], the isolate 386 was identified as* Streptomyces gramineus *[32] with 100% similarly (accession number HM748598.2); the isolate 73 had close similarity with two Actinobacterial strains:* Streptomyces avellaneus* [33] (accession number AB184413.1, 99.77% sequence similarity) and* Streptomyces aburaviensis *[34] (accession number AY999779.1, 99.77% sequence similarity), but, according to morphological studies, the isolate 73 was identified as* Streptomyces aburaviensis *[35]; and the isolate 519 was identified as* Streptomyces psammoticus* (accession number AB184554.2, 99.77% sequence similarity).

### 3.2. Effect of Carbon and Nitrogen Sources on Cytotoxic Activity

Five carbon and nitrogen sources were evaluated to increase the cytotoxic activity of extracts from three isolates; that is why the ISP 2 media was modified (8.0 g carbon source and 14 g of nitrogen source); the results for carbon source are shown in Tables 3 and 4. The carbon source to which the strain* Streptomyces aburaviensis* (73) had the higher cytotoxic percent was starch, obtaining cytotoxic values of 91.22%, 83.9%, and 68.1% at 100 ppm for prostate, lung, and breast cancer. Likewise, the values of IC_50_ for each cell line were 50.26 ± 2.54, 26.16 ± 3.26, and 26.65 ± 0.86 ppm, respectively, being more active for lung tumour cell line (A549). The strain* Streptomyces gramineus* (386) had the highest cytotoxic percent, with glucose as a carbon source for the PC3 tumour line and xylose for A549 and MDA-MB-231, obtaining values of 90.64%, 83.18%, and 65.63%, respectively. The strain* Streptomyces gramineus* (386) was more active for prostate tumour cell line (PC3) using glucose as a carbon source with an IC_50_ value of 11.18 ± 3.85 ppm. Finally, the strain* Streptomyces psammoticus* (519) had the highest cytotoxic percent at 100 ppm with xylose as the carbon source, obtaining values of cytotoxic percent at 100 ppm higher than 80%, and the values of IC_50_ were 55.80 ± 2.96, 48.58 ± 2.39, and 39.98 ± 3.27 ppm for prostate, lung, and breast cancer, respectively; this strain was more active for breast cancer (MDA-MB-231).

The results of the cytotoxic activity of different nitrogen sources in the ISP 2 broth are shown in Tables 4 and 5. The nitrogen sources that improved the cytotoxic activity for the isolate* Streptomyces aburaviensis* (73) were meat extract for the prostate tumour cell line and yeast extract for lung and breast cancer, obtaining cytotoxic percent of 77.14%, 63.45%, and 75.78% at 100 ppm, respectively. The IC_50_ values were measured using yeast extract as a nitrogen source, and the IC_50_ values were 74.36 ± 1.05, 69.13 ± 1.05, and 45.39 ± 3.06 ppm for prostate, lung, and breast tumour cell lines. The strain* Streptomyces gramineus* (386) had the best cytotoxic activity with malt extract with values of cytotoxic percent higher than 50% and had the highest cytotoxic activity for the prostate tumour cell line with an IC_50_ value of 14.59 ± 2.82 ppm. Furthermore, the strain* Streptomyces psammoticus* (519) had the best cytotoxic activity using malt extract as a nitrogen source with values of 49.13%, 59.73%, and 77.59% for lung, breast, and prostate cancer, respectively, and had the lowest IC_50_ for the breast tumour cell line with a value of 41.23 ± 2.84 (Table 4).

### 3.3. Media Optimization Using Central Composite Design

After establishing the carbon and nitrogen sources, the effects of the concentration of nitrogen source (A), carbon source (B), and pH (C) were studied using a central composite to determine the optimum level of these parameters leading to the maximum cytotoxic percent. The experimental design was tested carrying out 15 experiments for each isolate in the cell line that had the best cytotoxic effect; results are presented in Table 6.

One model was obtained from analysis of variance for each extract. F-test for the models showed statistical significance (P<0,05), indicating that all models fit and can adequately explain the variations observed in the cytotoxic percent. Moreover, the lack of fit was not significant in all models at the 5% level (P<0.05), indicating that the experimental data obtained fit well with the model [21]. Coefficients of correlation R^2^ for the isolates* Streptomyces aburaviensis *(73),* Streptomyces gramineus* (386), and* Streptomyces psammoticus* (519) were 0.8089, 0.8677, and 0.8020, respectively.

Table 7 shows the statistics analysis of cytotoxic activity from the isolate* Streptomyces gramineus* (386). As can be observed, the variables A, BC, and C^2^ were significant (P value<0,05). The regression equation for the model in terms of cytotoxic percent (Y) as a function of three independent variables (A: %w/v malt extract, B: %w/v glucose, C: pH) is given below.

(2) Y386=5,861+36,996A+2,260B+12,823C−5,3AC−0,516BC−0,277C2

**Figure 5:**
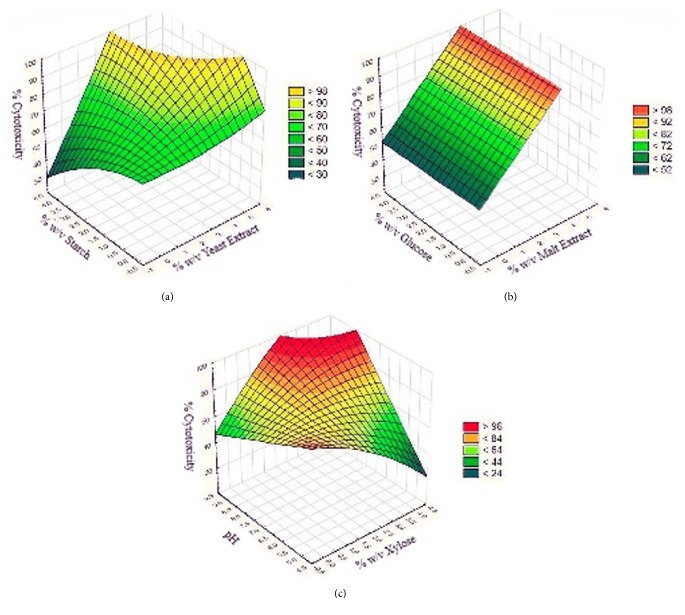
Surface plots for interactions between nitrogen source concentration and carbon source concentration at the best pH predicted for (a)* Streptomyces aburaviensis* (73) pH 6 and (b)* Streptomyces gramineus* (386) pH 7. The surface plot for (c) * Streptomyces psammoticus* (519) represents the interaction between carbon source concentration and pH with best nitrogen source concentration (1% w/v) predicted.

The response surface plot for the cytotoxic activity of* Streptomyces gramineus* (386) against lung cancer shows the interactive effect of different variables (%w/v glucose, %w/v malt extract, and pH) presented in Figure 5(b). The highest theoretical value to maximize the cytotoxic percent to 100% was with 5% of malt extract, 3% of glucose, and a pH value of 6.

The regression equation for the cytotoxic activity from the* Streptomyces aburaviensis *(73) is shown in (3), where Y is the response in terms of cytotoxic percent, and the variables were %w/v yeast extract (A), %w/v starch (B), and pH (C). The significant terms (P value<0,05) for this model are A, B, C, AB, AC y B^2^. In the response surface plot presented in Figure 5(a), it can be observed that the highest point to obtain 100% of cytotoxicity appeared at 4% of yeast extract, 3% of starch, and a pH value of 7.

(3) Y73=95,859−22,383A+0,546B−3,566C+3.5660AB+2,671AC+0,260A2−1,194B2

Finally, the model for the cytotoxic activity from the* Streptomyces psammoticus *(519) is given below as (4), showing the interactive effect between the variables %w/v malt extract (A), %w/v xylose (B), and pH (C). The significant terms (P value<0,05) for this model were B, C, BC y B^2^. The response surface plot presented in Figure 5(c) shows that the highest point for the isolate 519 against breast cancer is at 1% of yeast extract, 4% of starch, and a pH value of 8.

(4) Y519=104,110−0,071A−33,498B−4,890C+5,784BC−1,316B2

**Figure 6:**
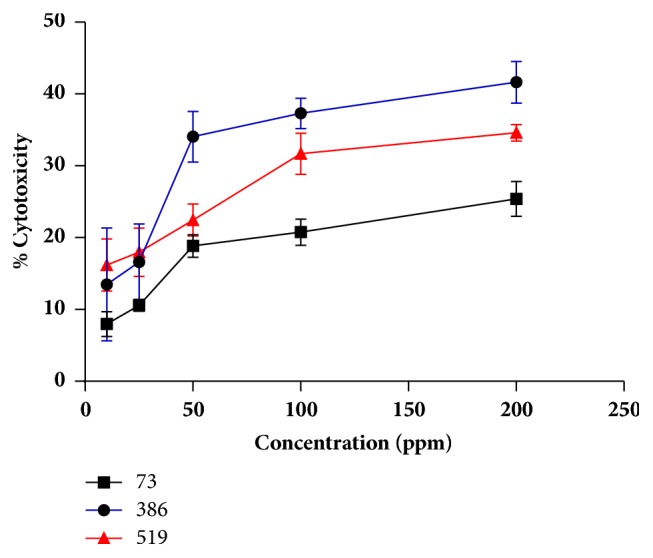
Cytotoxic activity tested in L929 cell line for* Streptomyces aburaviensis *(73),* Streptomyces gramineus* (386), and* Streptomyces psammoticus* (519). Standard deviation (n=3).

The best conditions of the tested variables obtained from the experimental design CCD had been verified experimentally (in triplicate) and compared with the predicted data (Table 8). The measured cytotoxic activity at 100 ppm was 89.29% ±4.05, 89.03% ±6.47, and 66.18% ±2.83 for* Streptomyces aburaviensis *(73),* Streptomyces gramineus* (386), and* Streptomyces psammoticus* (519), respectively. The predicted values from the model were 97.47%, 97.96%, and 95.89%. The verification revealed a high degree of accuracy of the model for* Streptomyces aburaviensis *(73) and* Streptomyces gramineus* (386), indicating the model's validation under the tested conditions. Moreover, the values of IC_50_ were measured at the best growth and nutritional conditions showing an increase compared with the initial results (Table 2). These assays were tested in L929 cell line without significative cytotoxic activity at low concentrations of extract (Figure 6).

### 3.4. Dry Biomass Growth Curve

**Figure 7:**
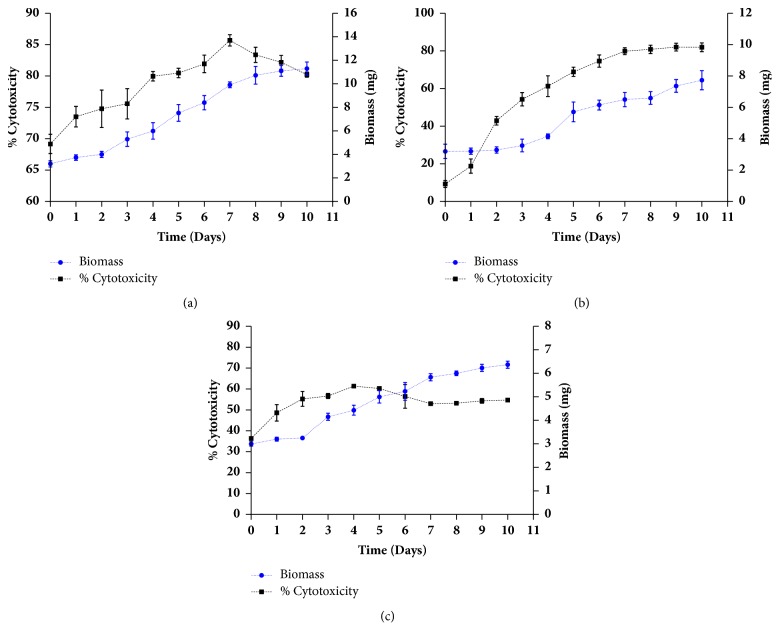
Growth kinetics of* Streptomyces aburaviensis* (73) against lung tumour cell line (a),* Streptomyces gramineus* (386) against prostate tumour cell line (b), and* Streptomyces psammoticus *(519) against breast tumour cell line (c) and dependent variables growth (mg; (blue filled circles)) and %cytotoxicity (%; (black filled squares)). Standard deviation (n=3).

The growth curves (Figure 7) obtained for this study were constructed from the average dry biomass obtained from three experiments after 10 days. The average of the dry biomass with standard error bars was plotted with the help of the statistical program GraphPad Prism® version 6.01. The Gompertz model (Table 9) was used to describe the sigmoidal microbial growth curves shown in Figure 7; all models exhibit a high correlations (R^2^) with values of 0.99 for* Streptomyces aburaviensis *(73), 0.98 for* Streptomyces gramineus *(386), and 0.98 for* Streptomyces psammoticus *(519).

### 3.5. LC-MS Analysis

**Figure 8:**
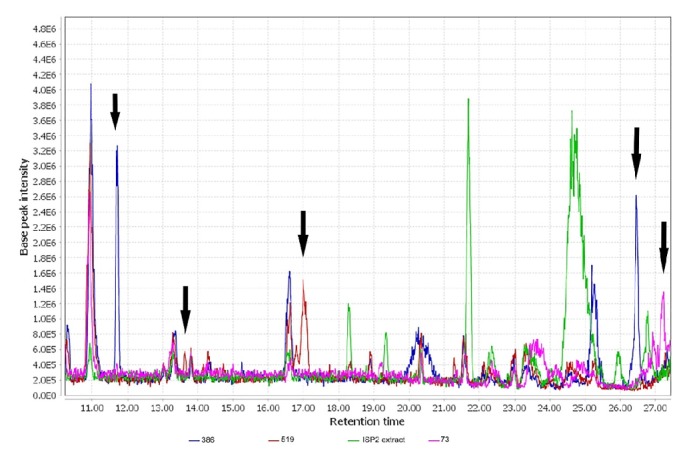
Liquid Chromatography comparison spectrum for organic extracts from* Streptomyces gramineus* (386),* Streptomyces aburaviensis* (73), and* Streptomyces psammoticus *(519) and control extract from ISP 2 media without biomass (the arrows indicate the peaks that differ from the other extracts).

The chromatograms obtained for the extracts tested showed defined signal patterns characteristic of each type of extract as indicated by arrows (Figure 8). Organic extracts showed the majority of signals between 11 and 28 min retention time (RT) and signals with mass/charge (m/z) relationship values from 160 to 820. The extract of* Streptomyces gramineus* (386) differs from the extracts of* Streptomyces aburaviensis* (73) and* Streptomyces psammoticus *(519) in the signals corresponding to retention time of 11.67 min, m/z= 408.6 and 26.44 min, m/z= 709.4. The strain* Streptomyces psammoticus *(519) presented characteristic signals at 13.62 min, m/z= 207.25 and 17.01 min, m/z= 453.2 and* Streptomyces aburaviensis* (73) at 27.25 min, m/z= 817.7.

## 4. Discussion

Cancer is the second leading cause of death in the world behind cardiovascular disease [5]. Despite this, chemotherapy is still one of the most used cancer treatments even with its range of serious side effects. To decrease the side effects of chemotherapy, new biologically active metabolites are needed to be explored, and natural product extracts continue to be the most promising source of new drugs for cancer [14]. Through this research, we found three* Streptomyces* strains isolated from Guaviare river sediments (Colombia, South America) with cytotoxic activity against prostate, lung, and breast cancer cell lines. The isolates were identified morphologically and molecularly as* Streptomyces aburaviensis *(73),* Streptomyces gramineus* (386), and* Streptomyces psammoticus* (519). Some studies describe* Streptomyces aburaviensis *(73) [35, 36] and* Streptomyces psammoticus* (519) [37, 38] with antibacterial and antifungal activity. In fact only* Streptomyces gramineus* (386) has been reported with cytotoxic activity; two compounds have been isolated from this strain with a moderate cytotoxic activity against SKOV-3 (ovarian cancer cell line), Meso-1 (human malignant mesothelioma cell line), and Jurkat (leukemia cell line) cells with IC_50_ values of 2.3, 2.5, and 1.0 *μ*M [39].

Three organic extracts were obtained from strains* Streptomyces aburaviensis *(73),* Streptomyces gramineus* (386), and* Streptomyces psammoticus* (519), which presented cytotoxic activity against lung, prostate, and breast cancer, respectively (Table 2). Furthermore, the organic extracts showed chemical differences analysed in LC-MS chromatogram (Figure 8).* Streptomyces gramineus* (386) spectrum showed a signal related to the compound diacarnoxide-A (RT=11.67, m/z= 408.6), which has been reported with cytotoxic activity against prostate cancer (PC3) and breast cancer (MDA-MB231) by Jingqiu et al. [40]. LC-MS analysis showed the possible presence of Streptophenazine I (RT=17.01; m/z= 453.2 (M+H)^+^) in the organic extract of* Streptomyces psammoticus* (519), which has been reported by Bunbamrung et al. with cytotoxic activity against breast (MCF-7) and lung cancer (NCI-H187) [41].

In order to optimize the cytotoxic activity of the three* Streptomyces* strains, five carbon sources were evaluated. Several reports take into consideration the role of the carbon source to enhance the biological activity, therefore* Streptomyces aburaviensis *(73) improved 2.58 times the cytotoxic activity against the lung tumour cell line using starch instead of glucose as a carbon source. Thumar et al. [42] demonstrated that the use of starch as a carbon source in* Streptomyces aburaviensis *(73) enhances growth and antibiotic production. Moreover,* Streptomyces gramineus* (386) had the best cytotoxic activity against the prostate cancer cell line using glucose as a carbon source instead of xylose because glucose demonstrated a higher cytotoxic activity at lower concentrations of extract. Similarly, Lee et al. [32] reported that glucose favours the growth of this strain as in this investigation.* Streptomyces psammoticus* (519) had the higher cytotoxic activity using xylose as a carbon source as Hamed et al. [43] reported that this strain is able to metabolize xylose to grow in culture, and Sujatha et al. [44] reported antibiotic production using this carbon source.

Subsequently, the nitrogen sources that increased the cytotoxic activity for* Streptomyces *strains were malt extract and yeast extract, which means that the cytotoxic activity is related to the synergic interaction between these nitrogen sources. It is why the cytotoxic screen was made using ISP 2 and the production of the cytotoxic compounds must be stimulated for these nitrogen sources. Likewise, some studies confirm that the nature of the nitrogen sources strongly affects bioactive compounds production [17] in different organisms. Raytapadar and Paul [45] found that the yeast extract supports the growth in* Streptomyces aburaviensis *(73). Similarly, the antibiotic [43] and laccase [46] production was enhanced using yeast extract as a nitrogen source in* Streptomyces psammoticus* (519), and the cytotoxic production was increased by yeast extract in* Streptomyces sp. VITPSA* [47].

The cytotoxic activity of the three* Streptomyces* strains was affected by different environmental and nutritional growth conditions (Figure 5). Firstly, the pH was the most influential variable in all models, the range 7–9 of pH constitutes the optimal values for* Streptomyces aburaviensis *(73) and* Streptomyces psammoticus* (519). Researchers confirm that* Streptomyces aburaviensis *(73) grow well at pH of 9 [42] and enhance its antibiotic production at 7.4 [45]. Moreover,* Streptomyces psammoticus* (519) increases the production of extracellular bioactive metabolites in a pH of 6.5 [43]. At last* Streptomyces gramineus* (386) has an optimal pH value of 6, and some studies report that this strain grows well at pH 4–8 [45]. Similarly, the interaction between starch with pH and yeast was significant to increase the cytotoxic activity of* Streptomyces aburaviensis *(73). The most important conditions besides pH for* Streptomyces gramineus* (386) and* Streptomyces psammoticus* (519) were the nitrogen and carbon sources, respectively.

Balachandran et al. [15] showed the relationship between cytotoxic activity and cell growth; this behaviour was observed in the present investigation for* Streptomyces aburaviensis *(73), which had a growth rate (*μ*_Max_) of 1.18 day^−1^ (Table 9) and had the maximum cytotoxic activity on the seventh day according to the inflection point of the growth curve (Figure 7(a)). Also, some studies of* Streptomyces aburaviensis *have reported that this strain secreted antibiotic in the culture after seven days of fermentation [45]. The strain* Streptomyces gramineus* (386) also had the highest cytotoxic activity in the seventh day, and it kept stable until the tenth day with a growth rate (*μ*_Max_) of 0.98 day^−1^ (Table 9). In addition, the strain* Streptomyces psammoticus* (519) had a growth rate (*μ*_Max_) of 0.50^day-1^ (Table 9) and presented the cytotoxic activity in the exponential phase. The cytotoxic activity (Figure 7(c)) of* Streptomyces psammoticus *(519) decreases when the stationary phase begins (fifth day); this behaviour is similar in comparison with Hamed-Mohamedin et al., who set the fermentation day until the fifth day for the production of bioactive metabolites using* Streptomyces psammoticus* [43].

Consequently,* Streptomyces aburaviensis *(73) and* Streptomyces gramineus* (386) at the best nutritional and growth conditions showed a high cytotoxic activity, and* Streptomyces psammoticus* (519) had a moderate cytotoxic activity. Despite this, the National Cancer Institute of United States estimated that the value of IC_50_ has to be lower than 30 ppm to have a high cytotoxic activity for a crude extract [48]. Hence, the value of IC_50_ for* Streptomyces aburaviensis (73) *against lung cancer (A549) was 25.00 ± 1.86 ppm, which was much greater than other* Actinobacteria*'s cytotoxic activities. For instance,* Nocardia dassonvillei* produced a novel anticancer phenazine that had a IC_50_ of 38.53 ppm against A549 [49]. Also, the Actinobacterial strains* ERIA-31* and* ERIA-33* produced a protease enzyme that was tested in A549 cell line and presented IC_50_ values of 57.04 ppm and 55.07 ppm, respectively [50].

*Streptomyces psammoticus* (519) had a IC_50_ value of 35.53 ± 2.7 ppm against breast cancer (MDA-MB-231). Similarly, Actinobacterial strains ACT01, ACT02, and ACT03 showed IC_50_ values of 32.79 ± 6.94, 69.84 ± 19.54, and 84.09 ± 18.93 ppm against MDA-MB-231 [16]. However, recently Actinobacterial strains EGY2 and EGY39 showed a high cytotoxic activity for the crude extracts with IC_50_ values of 19.50 ± 0.03 ppm and 29.6 ± 0.43 mg/mL, respectively [53]. Finally, the strain* Streptomyces gramineus* (386) showed the highest IC_50_ value among all three* Streptomyces* strains studied in this investigation, with a value of 7.93 ± 0.98 ppm against prostate cancer (PC3) with the growth and environmental conditions of the experimental design, but it does not have a significant difference with the first value of 6.14 ± 2.07 ppm (Table 2).

The results indicate that* Streptomyces aburaviensi*s (73),* Streptomyces gramineus* (386), and* Streptomyces psammoticus* (519) showed a promising cytotoxic activity against lung, prostate, and breast cancer, respectively. Furthermore, the extracts did not show a significative toxic activity against L929 (mouse fibroblasts) at values lower than 50 ppm of extract (Figure 6). The optimizing nutritional and growing factors improve the cytotoxic activity of* Streptomyces aburaviensis* (73) in 61.35% against lung cancer and 22.57% for* Streptomyces psammoticus* (519) against breast cancer.

## 5. Conclusions

The study revealed that the optimization of the environmental and nutritional conditions in growth culture improves the cytotoxic activity of extracts from* Streptomyces aburaviensis *(73) and* Streptomyces psammoticus* (519). However,* Streptomyces gramineus* (386) had a high cytotoxic activity (less than 30 ppm) with the initial conditions because the medium propitiated the stimulation of the cytotoxic activity without being able to improve it more.

This study reveals that the strategy of optimizing the environmental and nutritional conditions in growth culture had a positive impact in the cytotoxic activity, leading the investigation group to a new strategy. Also, the incorporation of techniques like HPLC-MS and MR will be used to identify the compounds responsible for the cytotoxic activity. In future studies, flow cytometry and Western blot will be carried out to identify the cellular and biochemical mechanisms that lead to the cytotoxic activity of extracts.

## Figures and Tables

**Table 1:** Cytotoxic activity of forty Actinobacterial extracts against prostate (PC3), lung (A549), and breast (MDA-MB-231) cancer lines (Table 1). NA: non-activity. The results are expressed as an average ± standard deviation (n=3).

**Strain**	**Cytotoxic Activity **(%)		
	**PC3**	**MDA-MB-231**	**A549**
4	NA	NA	13.05±2.08

5	3.11±5.21	20.2±3.75	13.5±5.18

11	3.06±3.57	18.5±4.31	13.1±3.57

17	3.85±4.12	21.1±2.97	6.9±2.78

18	NA	16.11±5.26	1.5±2.35

20	24.24±2.78	NA	10±6.43

26	35.44±2.97	24.2±4.82	4.7±2.54

28	50.46±3.3	58.3±5.32	9.9±1.59

29	6.98±23.65	35.7±5.11	NA

30	18.78±2.26	NA	51.5±4.72

32	76.5±5.82	61±4.73	36.21±4.62

41	18.57±2.49	22.5±5.65	37.32±3.38

42	16.7±3.86	46.3±6.76	38.2±5.33

44	13.48±1.27	7.035±4.98	NA

48	15.6±5.42	5.05±3.97	NA

73	88.21 ± 3.85	64.94±1.96	75.69±2.42

83	NA	2.8±4.48	0.7±3.85

105	49.2±4.21	46.5±5.64	56.32±3.49

159	15.86±2.39	30.6±2.37	26.9±5.32

171	31.94±1.76	49.8±4.84	24.9±4.39

194	2.78±3.12	16±7.11	NA

239	30.41±6.72	57.1±3.16	10.5±4.61

245	5.74±3.21	27.1±5.96	NA

259	34.2±3.31	17.31±4.64	37.02±5.83

278	30.93±2.98	16.82±0.12	57.11±4.74

294	16.87±4.98	66.6±2.34	1.46±3.76

339	47.41±6.27	36.6±4.72	3.1±5.56

363	27.86±3.45	53.5±4.61	3.5±4.42

386	89.73±2.07	59.28±2.67	78.12±3.01

389	15.12±4.21	39.2±4.16	23±5.35

424	7.72±3.08	49.7±6.23	1.6±3.78

429	21.12±2.73	35.7±4.97	1.4±3.34

431	30.42±4.84	39.7±3.52	20.21±3.16

441	NA	NA	39.1±4.56

452	2.1±3.21	NA	0.8±3.42

461	NA	5.5±6.95	7±4.21

487	5.21±3.2	NA	13.4±5.67

491	11.12±4.37	20±1.01	9.31±3.98

519	85.12±4.76	62.17±4.36	84.71±2.86

**Table 2:** Determination of IC_50_ values for the extracts of strains 73, 386, and 519 cultivated in ISP 2; against prostate (PC3), lung (A549), and breast (MDA-MB-231) tumour lines. The results are expressed as an average ± standard deviation (n=3).

**IC**_**50**_ **(ppm)**			
**Isolate**	**PC3**	**A549**	**MDA-MB-231**
**73**	64.84±3.85	67.69±2.42	43.24±1.96

**386**	6.14±2.07	33.17±2.67	70.72±4.87

**519**	64.58±4.76	60.54±2.86	45.89±2.36

**Table 3:** Determination of the carbon source that improved the cytotoxic percent at 100 ppm of the *Streptomyces* extract for prostate (PC3), lung (A549), and breast (MDA-MB-231) tumour lines. The results are expressed as an average ± standard deviation (n=3).

%** Cytotoxicity at 100 ppm**									
	**73**			**386**			**519**		
	**PC3**	**A549**	**MDA**	**PC3**	**A549**	**MDA**	**PC3**	**A549**	**MDA**
**Saccharose**	90.07 ± 0.21	70.31± 3.15	65.96± 3.06	89.81± 0.5	75.11± 0.74	65.1± 3.61	88.71± 0.82	85.18± 0.18	59.18± 0.84

**Glucose**	89.39 ± 0.63	78.17± 2.54	63.27± 1.62	90.64± 0.56	80.69± 1.38	57.11± 2.03	89.10± 0.67	86.78± 2.86	66.17± 1.83

**Maltose**	89.05 ± 1.05	76.09± 0.89	57.86± 2.74	87.64± 1.86	72.28± 1.24	61.33± 1.07	89.70± 0.48	86.61± 4.87	59.27± 0.72

**Xylose**	89.97 ± 1.77	81.83± 4.76	65.69± 3.01	89.60± 0.57	83.18± 1.63	65.63± 2.32	91.12± 1.02	87.78± 1.26	74.76± 2.79

**Starch**	91.22 ± 0.08	83.90± 1.05	68.10± 1.24	88.93± 1.02	81.49± 0.38	64.35± 0.84	90.59± 0.22	87.79± 0.58	62.58± 0.67

**Table 4:** Determination of the IC_50_ using the best carbon and nitrogen sources in *Streptomyces* strains for prostate (PC3), lung (A549), and breast (MDA-MB-231) tumour lines. The results are expressed as an average ± standard deviation (n=3).

**IC**_**50**_				
**Isolate**	**PC3**	**A549**	**MDA-MB-231**	**Carbon source/ Nitrogen source**
**73**	50.26± 2.54	26.16± 3.26	26.65± 0.86	Starch
	74.36± 1.05	69.13± 1.05	45.39± 3.06	Yeast Extract

**386**	11.18± 3.85	31.64± 1.45	68.57± 1.08	Xylose
	14.59± 2.82	16.35± 4.03	71.25± 1.72	Malt Extract

**519**	55.80± 2.96	48.58± 2.39	39.98± 3.27	Xylose
	68.43± 2.74	50.49± 3.64	41.23± 2.84	Malt Extract

**Table 5:** Determination of the nitrogen source that improved the cytotoxic percent at 100 ppm of the *Streptomyces* extract for prostate (PC3), lung (A549), and breast (MDA-MB-231) tumour lines. The results are expressed as an average ± standard deviation (n=3).

%** Cytotoxicity at 100 ppm**									
	**73**			**386**			**519**		
	**PC3**	**A549**	**MDA**	**PC3**	**A549**	**MDA**	**PC3**	**A549**	**MDA**
**Yeast extract**	75.86± 0.35	63.45± 0.65	75.78± 0.61	69.32± 0.28	23.18± 3.96	18.07± 2.70	70.9 ±0.92	37.86± 0.79	46.49 ±0.80

**Malt extract**	68.72± 0.4	22.50± 0.59	33.24± 0.03	86.76± 4.76	67.93± 5.72	54.46± 0.36	77.59± 1.2	49.13± 2.55	59.73 ±2.16

**Meat extract**	77.14± 1.5	33.69± 5.58	48.85± 0.02	72.93± 0.98	21.82± 0.85	29.77± 1.54	68.42± 0.56	40.91± 2.96	47.46 ±1.96

**Peptone**	71.12± 5.32	29.13± 5.93	37.59± 0.94	68.79± 3.92	28.56± 5.51	48.00± 0.98	64.92± 4.98	33.96± 4.81	33.96 ±2.83

**Potassium nitrate**	67.51± 3.75	16.07± 4.15	22.91± 1.87	66.39± 1.81	33.10± 1.93	37.28± 1.79	71.80± 3.74	31.18± 3.62	31.18 ±0.08

**Table 6:** Experimental design central composite design representing the cytotoxic percent by *Streptomyces *strains influenced by four variables and five levels of each on A: carbon source concentration, B: nitrogen source concentration, and C: pH.

**Run**	**Independent variables**			%** Cytotoxicity (100 ppm)**					
				**Observed value**			**Predicted Value**		
	**A**	**B**	**C**	**73 **	**386 **	**519 **	**73 **	**386 **	**519 **
	**(**%**w/v)**	**(**%**w/v)**		**Against PC3**	**Against A549**	**Against MDA**	**Against PC3**	**Against A549**	**Against MDA**
1	2.5	2	6	78.05	83.482	73.204	77.319	84.139	71.740

2	2.5	0.4	8	75.075	74.138	72.774	73.985	76.455	69.707

3	0.5	2	8	71.182	82.695	87.936	70.728	84.274	85.239

4	0.5	0.4	6	75.075	75.54	74.374	75.060	75.090	75.003

5	0.1	0.8	7	74.44	81.127	72.715	73.952	80.956	74.620

6	5	0.8	7	76.138	81.127	72.715	76.401	80.439	74.271

7	1.4	0.1	7	70.717	81.705	72.834	70.258	81.766	70.462

8	1.4	4	7	72.547	77.078	75.952	72.753	76.486	76.688

9	1.4	0.8	5	72.547	77.078	75.952	72.044	77.487	75.054

10	1.4	0.8	9	74.824	83.369	72.434	74.720	81.934	74.001

11	1.4	0.8	7	73.085	80.37	73.929	73.382	80.819	74.528

12	1.4	0.8	7	70.717	80.078	72.709	73.382	80.819	74.528

13	1.4	0.8	7	72.767	81.494	74.946	73.382	80.819	74.528

14	1.4	0.8	7	72.767	81.568	72.918	73.382	80.819	74.528

15	1.4	0.8	7	74.198	82.272	74.033	73.382	80.819	74.528

**Table 7:** Analysis of variance for the fit second order polynomial model and lack of fit for the cytotoxic percent by composite central design for the strain *Streptomyces gramineus* (386). SS: sum of squares, DF: degrees of freedom, MSS: mean sum of squares.

Source	Sum of squares	DF	Mean square	F value	p-value
Model	101.406	6	16.901	8.746	0.0037

A	0.162	1	0.162	0.084	0.7790

B	18.572	1	18.572	9.611	0.0147

C	32.920	1	32.920	17.037	0.0033

AC	90.358	1	90.358	46.762	0.0001

BC	0.644	1	0.644	0.333	0.5795

C∧2	1.987	1	1.987	1.028	0.3402

Residual	15.458	8	1.932		

Lack of Fit	12.147	4	3.037	3.669	0.1180

Pure Error	3.310	4	0.827		

Cor Total	116.865	14			

**Table 8:** Verification of the accuracy of the models obtained with CCP and de IC_50_ at the best environmental and growth conditions. Experimental results are expressed as an average ± standard deviation (n=3).

**Isolate**	%**Cytotoxicity at 100 ppm**	**Predicted**	**%Error**	**IC**_**50**_ **(ppm)**	**Cell line**
**73**	89.29% ±4.05	97.47	8.40%	25.00±1.86	A549

**386**	89.03%± 6.47	95.45	6.73%	7.93±0.98	PC3

**519**	66.18%±2.83	94.96	30.31%	35.53±2.71	MDA-MB-231

**Table 9:** Results of Gompertz model growth rate (*μ*Max), the lag time (*λ*), and the coefficients of correlation (R^2^).

**Isolate**	***μ***_**Max**(**day**)_	***λ*** ** (day)**	**R** ^2^
**73**	1.18	1.71	0.99

**386**	0.98	2.64	0.95

**519**	0.50	1.26	0.98

## Data Availability

The data used to support the findings of this study are available from the corresponding author upon request.
